# A high-selectivity 24-GHz SIW–DGS–CPW bandpass filter with wide stopband rejection for automotive radar and ADAS

**DOI:** 10.1038/s41598-026-41312-w

**Published:** 2026-03-23

**Authors:** Alaa M. Abada, Anwer S. Abd El-Hameed, Angie R. Eldamak, Hadia M. El-Hennawy

**Affiliations:** 1 Electronics & Communications Engineering Department, Ain Shams University, Cairo, Egypt; 2 Electronics Research Institute, Cairo, Egypt

**Keywords:** 24 GHz, ADAS, Automotive radar, Microstrip bandpass filter, Mm, Wave, SIW, DGS, CPW, Thermal analysis

## Abstract

A performance-efficient compact, highly selective 24 GHz SIW-based bandpass filter (BPF) is proposed for automotive radar and advanced driver-assistance systems (ADAS). The key contribution is a single-layer SIW–DGS–CPW co-design in which SIW cavities provide a high-Q passband, an open-rectangular DGS introduces transmission zeros to steepen the skirts and reinforce the stopband, and a CPW feeding transition improves matching and practical integration. Implemented on a substrate of Rogers RO4003C with ${\varepsilon }_{r}=3$ and thickness of 0.508 mm, the prototype occupies $4.27{\lambda }_{g}\times 2.14{\lambda }_{g}$, achieving a pronounced miniaturization while maintaining strong spectral selectivity. Full-wave simulations and measurements confirm a center frequency of 23.97 GHz and a 450-MHz 3-dB bandwidth, with return loss better than 24 dB and an in-band insertion loss of 1.6–2.0 dB (${S}_{21}$). The filter exhibits sharp roll-off with a measured 40-dB rejection within ± (0.8–1.2) GHz from ${f}_{0}$, and 30–35 dB suppression across 20–30 GHz. A compact equivalent-circuit model captures the passband behavior and transmission zeros. Thermal analysis (25–105 °C) shows only a slight downshift (~ 30 MHz) with minimal performance degradation, supporting automotive reliability. Compared with prior 24-GHz BPFs, the proposed co-integration simultaneously improves skirt selectivity and wide stopband suppression within a compact footprint.

## Introduction

The rapid evolution of autonomous driving and intelligent transportation systems has underscored the critical role of high-precision sensing platforms, particularly automotive radar and ADAS^1,2^. Operating within the mm-Wave spectrum, these systems demand passive components, most notably BPFs that exhibit high selectivity, low insertion loss, and a compact footprint suitable for dense RF integration^3^. Among the various allocated mm-Wave frequency bands, the license-free 24 GHz band stands out for its favorable trade-offs in resolution, range, and power efficiency, making it a widely adopted standard for short- and medium-range automotive radar^4^^,^^5^.

In high-frequency systems such as 24 GHz radar front ends, the performance of the BPF directly impacts system-level metrics including detection range, angular resolution, and electromagnetic compatibility (EMC)^6^. Filters must confine the operating band sharply while attenuating spurious harmonics and adjacent channels, all within stringent size and integration constraints. Recent microstrip and SIW BPFs around the 24–30 GHz range report diverse trade-offs in bandwidth, loss, and selectivity^7^. Representative examples are summarized as follows^8^ proposed a diagonally coupled SIW BPF with a compact footprint, though it suffered from a measured insertion loss of approximately 2.1 dB unsuitable for precision radar modules. Similarly, a dual-hole coupled SIW structure in^9^ demonstrated promising simulated behavior near 24 GHz, yet lacked fabricated validation, limiting its practical relevance^10^ proposed a compact wideband SIW-based bandpass filter operating across X, Ku, and K bands, achieving low insertion loss and good return loss. Although effective for wideband applications, the design does not utilize DGS or target 24 GHz automotive radar applications, highlighting the need for more selective structures in mmWave ADAS systems., though they exhibited significant size overhead and suboptimal passband alignment^11^. A triangular-slot SIW–DGS filter^12^ offered improved miniaturization and simulated stopband suppression; nonetheless, it did not include measured S-parameters, leaving its practical applicability uncertain.

Overall, recent 24-GHz and near-band SIW filters have advanced compactness and selectivity; however, the surveyed literature indicates that SIW resonators, DGS perturbations^13^, and planar feeding transitions are most often treated separately or in partial combinations, whereas a measurement validated single-layer co-integration of these elements for a 24 GHz automotive-radar BPF remains limited^14^. This motivates the proposed SIW–DGS–CPW co-design to simultaneously enhance skirt selectivity and reinforce wide stopband suppression within a compact footprint^15^. In^14^, a high-selectivity 24 GHz absorber demonstrated both transparency in the passband and broadband absorption, achieving a measured insertion loss of only 1.98 dB at 23.8 GHz. However, this approach relies on absorption mechanisms rather than true signal transmission, which may limit its use in active radar front ends. Meanwhile,^16^ proposed a tunable dual-band BPF using a stub-loaded dual-mode resonator operating at 23.92 and 28.38 GHz. Although their design supports bandwidth reconfiguration and hybrid radar/5G functionality, the reported insertion losses (~ 1.98 dB) and physical dimensions (~ 1.23 λg × 2.02 λg) remain suboptimal for compact, low-loss automotive radar applications at 24 GHz.

This paper presents a high-selectivity SIW-based bandpass filter, an open-rectangular DGS, and a CPW feed on a single-layer substrate of Rogers RO4003C (εᵣ = 3) with a footprint of (18 × 36) mm^2^. The filter exhibits a measured center frequency of 23.97 GHz with a 3 dB bandwidth of 450 MHz, optimized for short-range automotive radar. Experimental results confirm excellent performance, achieving an insertion loss of 1.62 dB and a return loss exceeding –24.25 dB (VSWR ≈ 1.03). A sharp roll-off rate of 53.13 dB/GHz and strong out-of-band rejection ensure spectral purity, while the group delay remains nearly flat, peaking at only 2.3 ns supporting low-distortion signal propagation. Also, the proposed filter achieves ${\mathrm{Q}}_{\mathrm{L}}\approx 53.3$, which is competitiveness with recent 24-GHz SIW-based designs while offering enhanced selectivity due to the introduced transmission zeros*.* Full-wave CST simulations show good agreement with measurements. Moreover, thermal analysis confirms high frequency stability under elevated temperatures, validating the filter’s robustness and reliability for next-generation ADAS applications.

## Filter design, modeling, and experimental validation

### Structural design and material specification

**Fig. 1:**
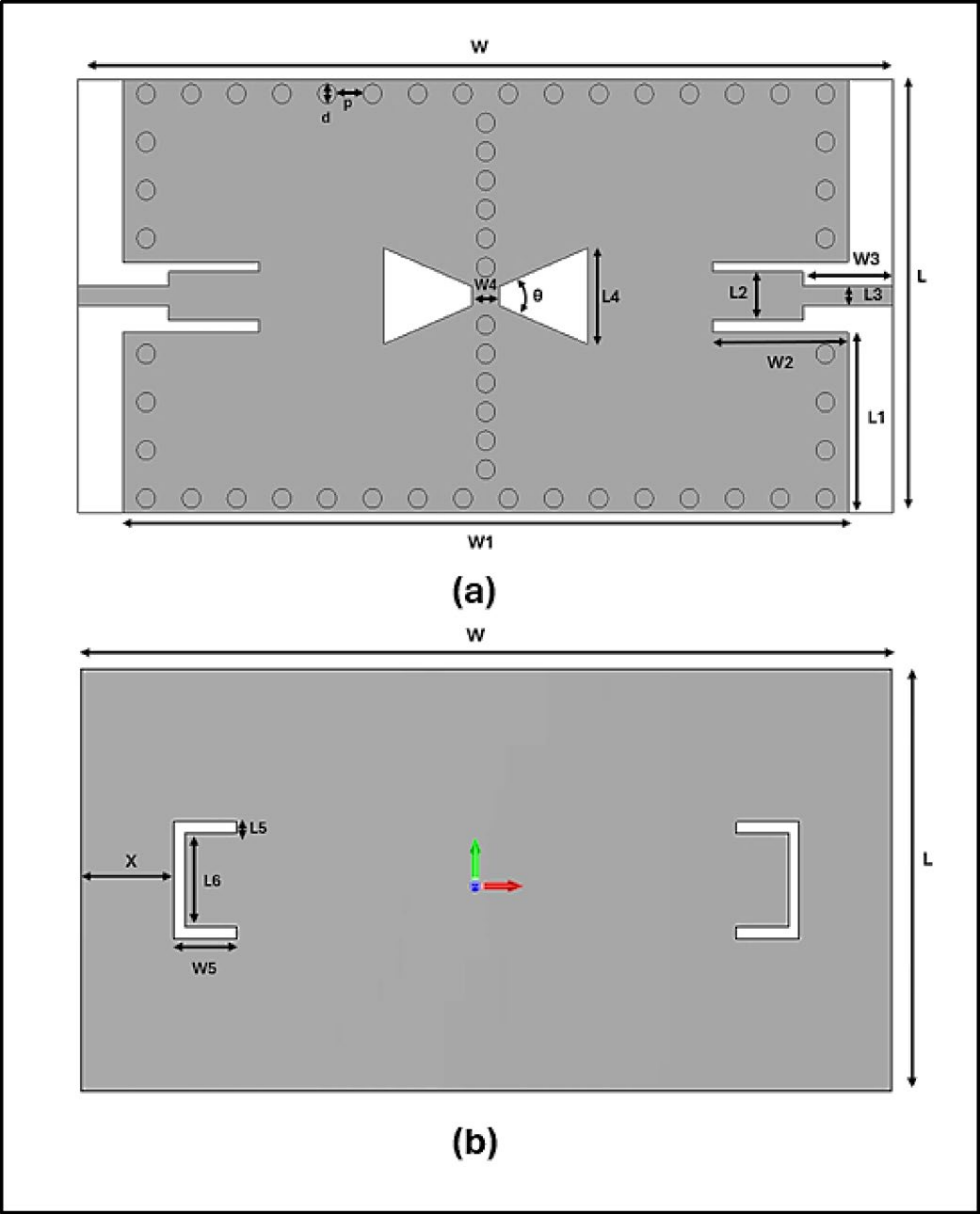
The proposed BPF design (**a**): front view, (**b**): back view.

Bandpass filters at mm-wave frequencies are fundamental to automotive radar front ends, where high selectivity, low insertion loss, and compact integration are essential^17^. To meet these stringent requirements, the proposed filter is developed through the structural advantages of SIW with the spectral shaping capabilities of a top-layer bowtie slot, Open Rectangular DGS on bottom layer, and CPW-fed excitation for efficient broadband coupling^18^. The proposed filter structure is implemented on a single-layer dielectric substrate with relative permittivity εᵣ=3 and thickness 0.508 mm, occupying a compact area of (18 × 36) mm^2^ as in Fig. 1. The effective dimensions of the proposed 24 GHz PBF cavity can be determined using equations (1) and (2), while the resonant frequency of the TE₁₀ₘ mode (in Hz) is given by equation (3). The physical dimensions of each section, including SIW width, via spacing, slot angles, and DGS lengths, are detailed in Table 1.

1 $${{\boldsymbol{L}}}_{{\boldsymbol{e}}{\boldsymbol{f}}{\boldsymbol{f}}}={\boldsymbol{L}}-1.08\frac{{{\boldsymbol{d}}}^{2}}{{\boldsymbol{p}}}+0.1\frac{{{\boldsymbol{d}}}^{2}}{{\boldsymbol{L}}}$$

2 $${{\boldsymbol{W}}}_{1{\boldsymbol{e}}{\boldsymbol{f}}{\boldsymbol{f}}}={\boldsymbol{L}}-1.08\frac{{{\boldsymbol{d}}}^{2}}{{\boldsymbol{p}}}+0.1\frac{{{\boldsymbol{d}}}^{2}}{{{\boldsymbol{W}}}_{1}}$$

3 $${\boldsymbol{F}}_{{{\boldsymbol{TE}}10{\boldsymbol{m}}}} = \frac{{{\boldsymbol{c}}_{0} }}{{2\sqrt {{\boldsymbol{\varepsilon}}_{{\boldsymbol{r}}} } }}\sqrt {\left( {\frac{1}{{{\boldsymbol{L}}_{{{\boldsymbol{eff}}}} }}} \right)^{2} + \left( {\frac{{\boldsymbol{m}}}{{{\boldsymbol{W}}_{{1{\boldsymbol{eff}}}} }}} \right)^{2} }$$

where c_0_ is the speed of light in free space, and *m* is the mode index in the longitudinal direction (m0<), representing the number of half-wavelengths along the cavity’s length.

**Table 1:** Dimensions of design.

Parameter	Value (mm)	Parameter	Value (mm)
W	36	L	18
W_1_	32	L_1_	7.6
W_2_	6	L_2_	1.0
W_3_	4	L_3_	0.8
W_4_	0.8	L_4_	4
W_5_	2.8	L_5_	0.5
X	4.15	L_6_	4
d	0.8	P	1.2

### Sequential development of the filter architecture

**Fig. 2:**
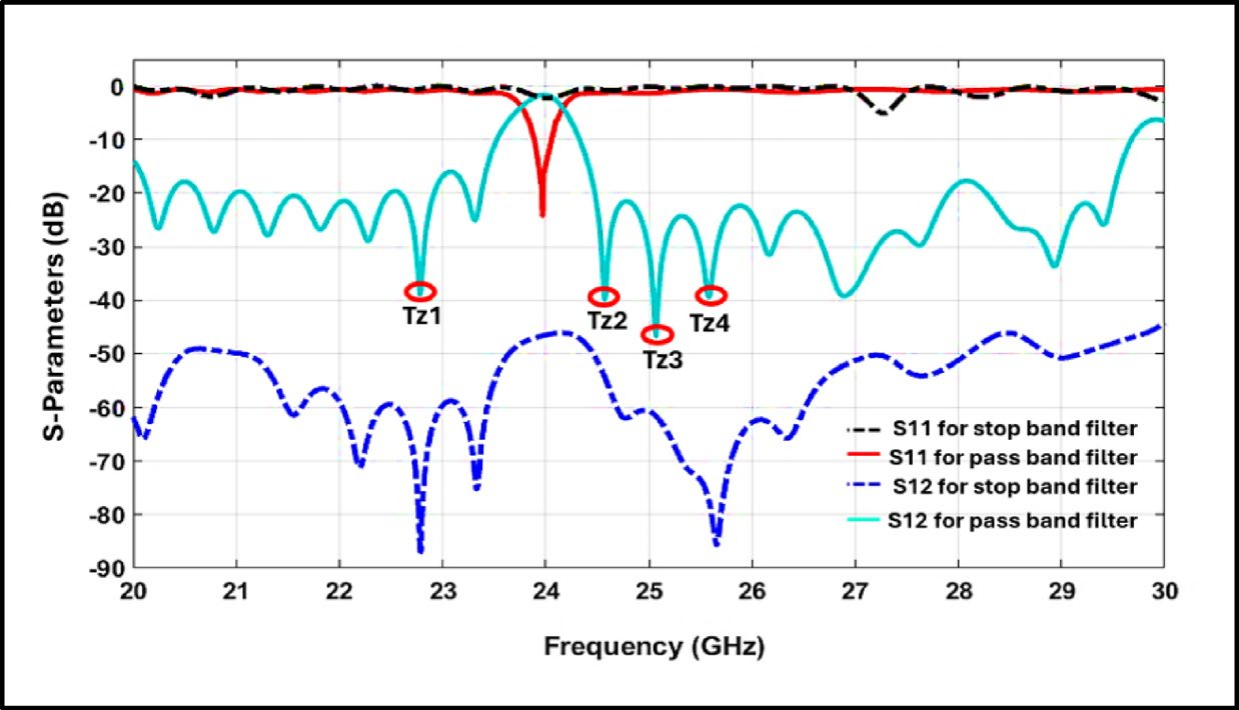
Scattering parameter for SPF filter and BPF.

The proposed filter was developed through a two-stage process that strategically integrates multiple microwave techniques to enhance spectral performance within a compact footprint. In the first stage, a Stopband Filter (SBF) was realized using a SIW cavity with CPW-fed input and output ports. A dedicated row of metallized vias was placed between the ports to suppress undesired coupling and ensure electromagnetic isolation^19^. The simulation results in Fig. 2 demonstrated a band-stop response, with S_12_ below –50 dB and high ${\mathrm{S}}_{11}$ across 20–30 GHz, confirming strong port isolation and the absence of resonance.

**Fig. 3:**
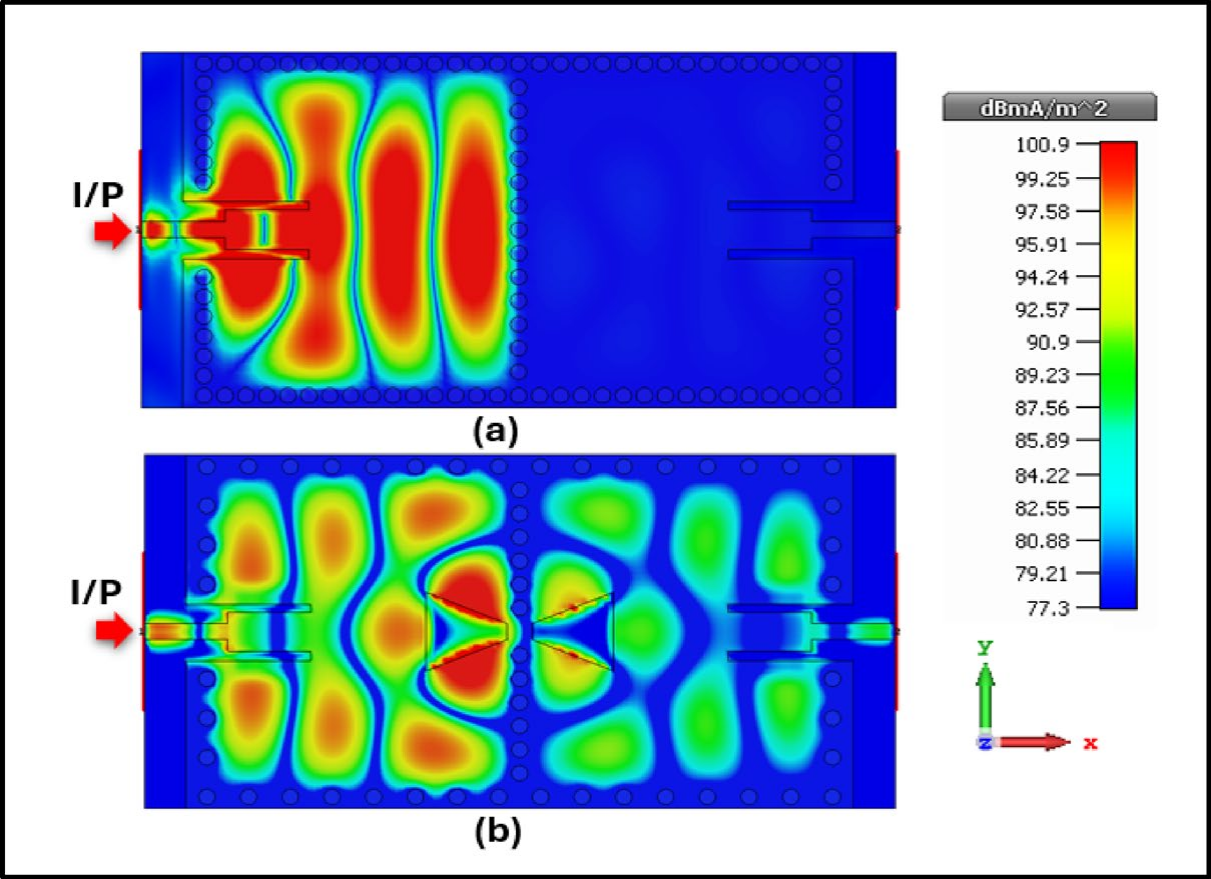
Currant density for (**a**) Stopband filter, (**b**) Band-pass filter.

In the second stage, a bowtie structure was defected on the top layer, significantly improving frequency selectivity around 24 GHz by sharpening the passband edge. Simultaneously, an Open Rectangular DGS was implemented on the bottom layer to shape the stopband response and introduce additional transmission zeros^20^^,^^21^. Figure 2 describes the difference in frequency response between the SBF and PBF. Figure 3 describes the surface current distribution for both configurations to evaluate the field confinement and resonance behavior, where (a) corresponds to the stopband filter stage and (b) represents the final passband filter after integrating the bowtie and DGS structures.

## Sensitivity analysis of filter design parameters

### Impact of coupling slot variation on filter response

**Fig. 4:**
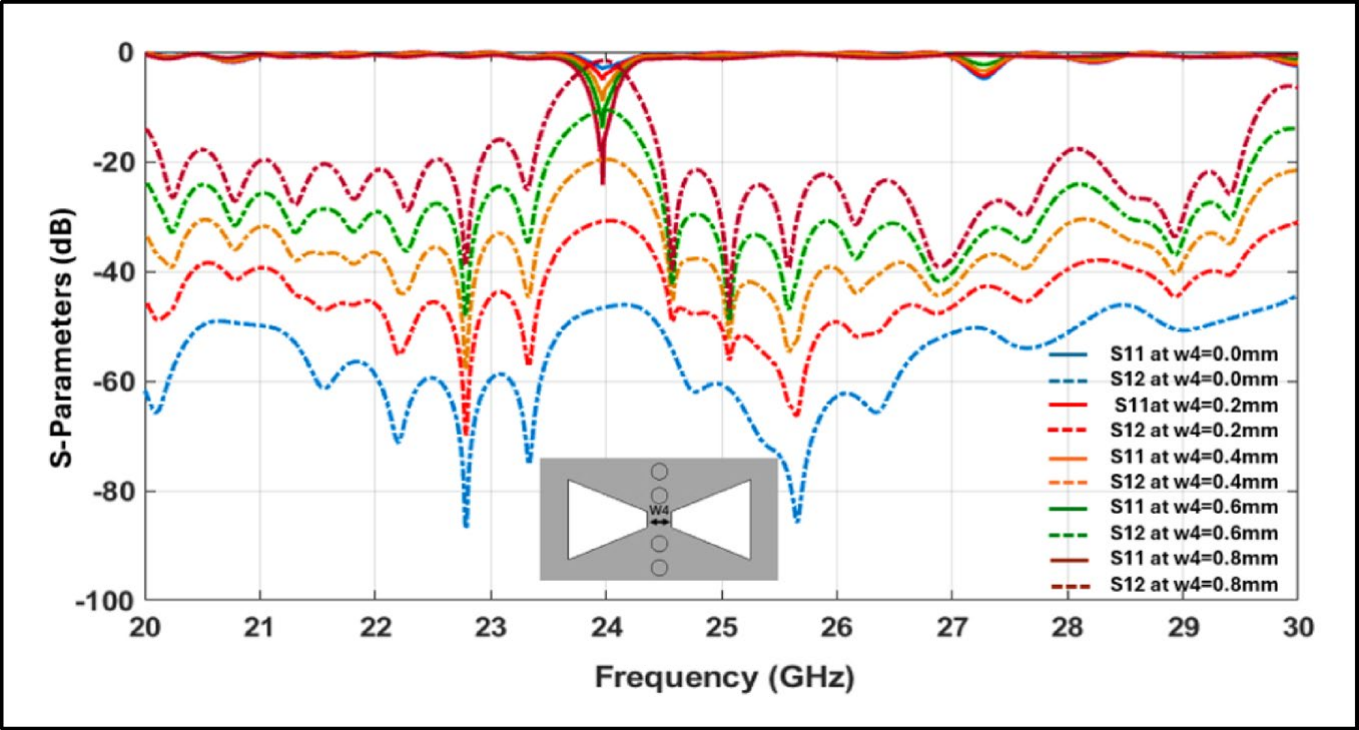
Frequency response of S₁₁ and S₁₂ for different coupling levels.

The simulated frequency responses of ${\mathrm{S}}_{11}$ and ${\mathrm{S}}_{21}$ under varying coupling conditions clearly highlight the filter’s sensitivity to structural variations^22^. In the proposed design, the coupling strength is primarily governed by the physical parameter W_4_, which determines the spacing between the resonant elements, as illustrated in Fig. 4. Variations in W_4_ result in observable shifts in the resonant frequency, return loss, and insertion loss, emphasizing the critical influence of coupling geometry on bandwidth tuning and overall filter selectivity^23^. This behavior is fundamentally attributed to changes in magnetic distribution and current density across the coupling aperture, which directly affects energy transfer efficiency between the SIW cavities^24^.

### Impact of substrate height variation on filter response

To evaluate how substrate thickness influences the electromagnetic behavior of the filter, a series of parametric simulations were performed using CST Studio Suite^25^. The study considered four substrate thicknesses: h_1_=1.527mm, h_2_=0.762mm, h_3_=0.508mm, and h_4_=0.254mm while maintaining a constant relative permittivity of ε_r_=3^26^.

**Fig. 5:**
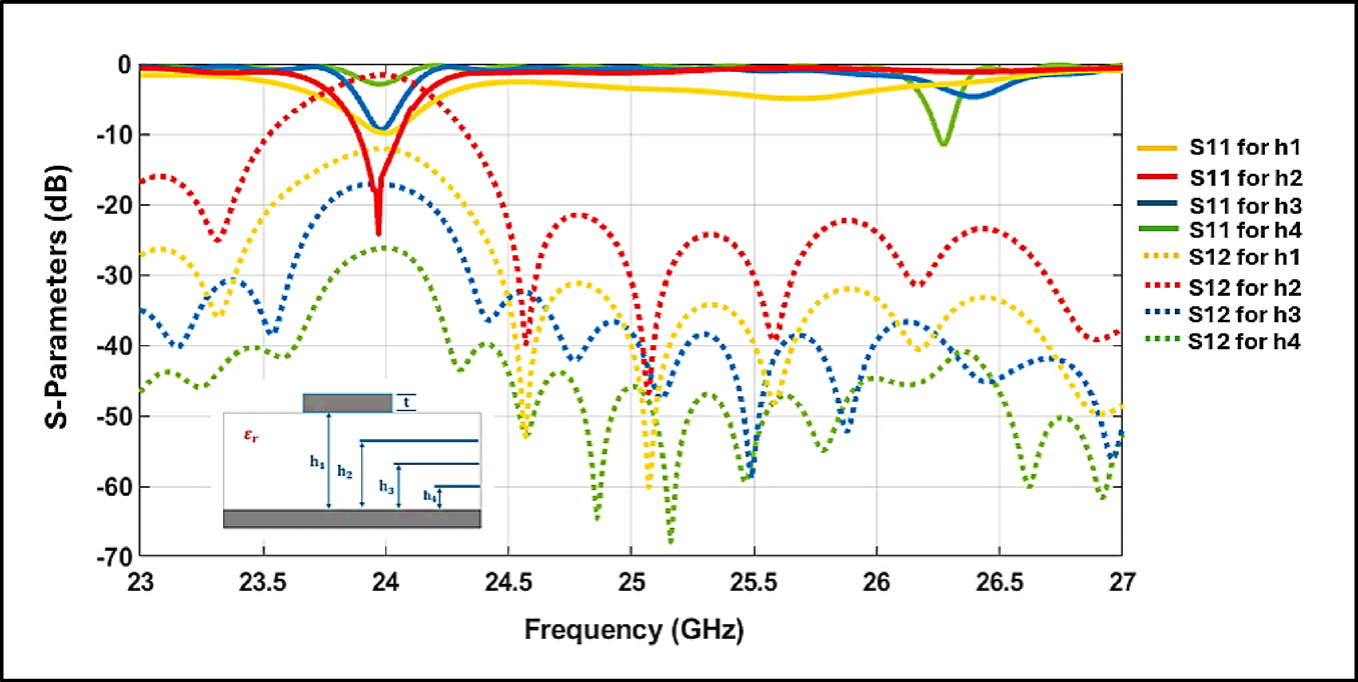
Simulated S-Parameters responses of the proposed BPF for different substrate thicknesses.

The simulated results, illustrated in Fig. 5, show that increasing substrate thickness causes a downward shift in the resonant frequency and slight degradation in return loss. These effects are attributed to changes in the distributed electromagnetic fields and are further supported by theoretical modeling. The equivalent capacitance C_eq_ and inductance L_eq_ of the SIW cavity vary as functions of the substrate thickness h, and can be approximated as in Eq. (4)^27^:

4 $$\begin{gathered} \frac{{{\boldsymbol{\mu}}_{0} .{\boldsymbol{h}}}}{{{\boldsymbol{W}}_{{1{\boldsymbol{eff}}}} }} \propto \user2{Leq ,} \hfill \\ \frac{{W_{1eff} .\varepsilon_{r} }}{h} \propto Ceqq \hfill \\ \end{gathered}$$

As h increases, L_eq_ increases due to expanded magnetic field loops, while C_eq_ decreases as the electric field confinement weakens. Among all configurations, a thickness of 0.508 mm was found to offer the optimal trade-off between L_eq_ and C_eq_, resulting in a sharp, stable, and well-controlled bandpass response centered at 24 GHz.

### Design rationale and parametric validation of SIW–DGS–CPW integration

**Fig. 6:**
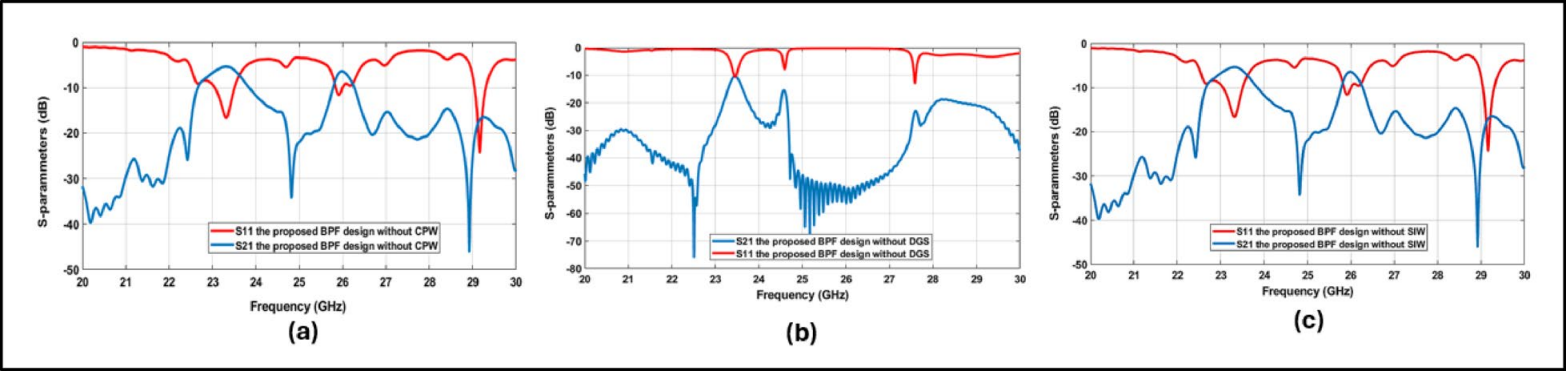
Parametric validation of the CPW–DGS–SIW integration: (**a**) without CPW feeding transition, (**b**) without DGS, and (**c**) without SIW.

To elucidate the electromagnetic role of each building block in the proposed topology, parametric validation was conducted by selectively removing one element at a time, as depicted in Fig. 6. When the CPW feeding transition is omitted (Fig. 6a), the filter exhibits degraded impedance matching and increased passband ripple, highlighting the critical role of the CPW transition in controlling external coupling and ensuring stable in-band matching. In the absence of the DGS (Fig. 6b), the transmission zeros near the passband edges vanish, and the wide stopband suppression is significantly weakened, confirming that the DGS is the dominant mechanism responsible for transmission-zero generation and skirt steepening. Conversely, removing the SIW via-fence structure (Fig. 6c) leads to poor field confinement, distorted resonant behavior, and reduced selectivity, demonstrating that the SIW cavities establish the fundamental passband resonance and bandwidth definition^28^. When the CPW feeding, DGS, and SIW cavities are co-integrated in the proposed design, these mechanisms act synergistically to achieve the reported high selectivity, controlled bandwidth, and strong out-of-band rejection required for 24-GHz automotive radar and ADAS applications.

## Lumped-element circuit modeling and analytical validation

An equivalent circuit model was developed using ABCD matrix theory to analytically characterize the filter’s electromagnetic performance^29^. The ABCD parameters of the series inductors, shunt capacitors, and coupling elements were cascaded to form the overall transmission matrix, which was then transformed into S-parameters assuming a reference impedance of 50 Ω as in equations (5) and (6).

5 $$\begin{gathered} T = \left[ {\begin{array}{*{20}c} A & B \\ C & D \\ \end{array} } \right] \hfill \\ S_{11} = \frac{{A + \frac{B}{{Z_{0} }} - \frac{C}{{Z_{0} }} - D}}{{A + \frac{B}{{Z_{0} }} + CZ_{0} + D}} \hfill \\ \end{gathered}$$

6 $${S}_{21}=\frac{2}{A+\frac{B}{{Z}_{0}}+C{Z}_{0}+D}$$

Each reactive element inductors, capacitors, and the coupling slot was represented by a corresponding lumped component that mirrors its physical function in the actual structure^30^.

In the Equivalent circuit, L_1_ and L_2_ model the series effects of the finite-length feeding/transition paths from the ports to the SIW section (magnetic energy storage along the current path). The shunt capacitors C_1_ and C_2_ represent the fringing electric-field capacitances created by the feeding/CPW transition gaps and discontinuities to the ground reference at the input and output, which largely govern port matching and external coupling. The series capacitor C_n_ corresponds to the dominant inter-resonator (inter-cavity) coupling across the central coupling iris/gap between the two SIW resonant sections; thus, it primarily controls the coupling strength and consequently the 3-dB bandwidth and skirt behavior.

**Fig. 7:**
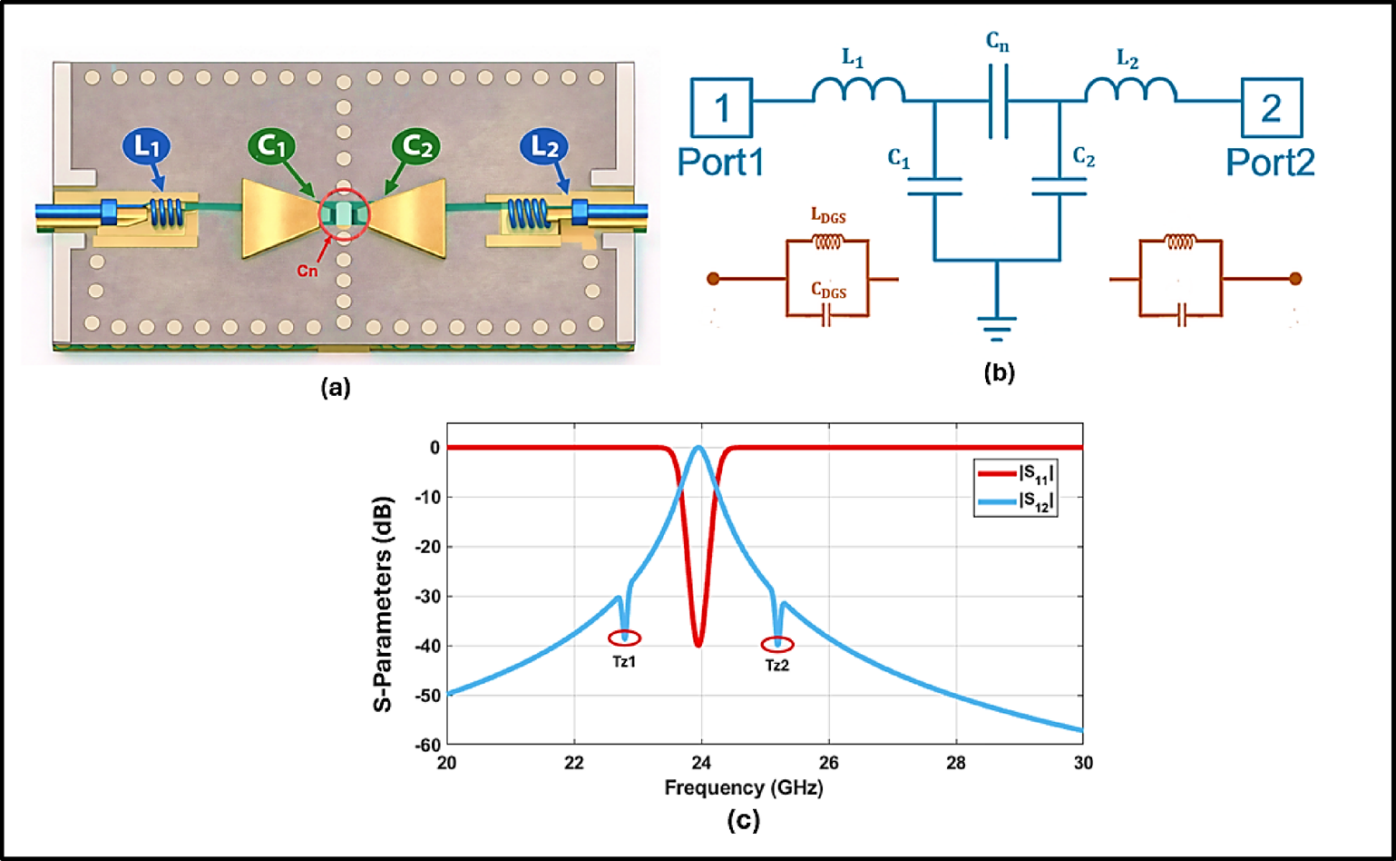
(**a**) Physical mapping of the equivalent-circuit elements onto the proposed SIW–DGS–CPW filter topology. (**b**) Equivalent circuit model of the proposed BPF. (**c**) Simulated S-Parameters using Keysight ADS.

Figure 7a provides a one-to-one physical mapping between the front-end lumped elements and their locations on the SIW–DGS–CPW topology. In the equivalent circuit of Fig. 7b, ${L}_{1}$ and ${L}_{2}$ model the series inductive effect of the finite-length feeding/transition paths from the ports to the SIW section (magnetic energy storage along the current path). The shunt capacitors ${C}_{1}$ and ${C}_{2}$ represent the fringing electric-field capacitances created by the CPW/feeding transition gaps and discontinuities to the ground reference at the input and output, governing port matching and external coupling. The series capacitor ${C}_{n}$ corresponds to the dominant inter-resonator electric coupling across the central coupling iris/gap between the two SIW resonant sections; therefore, it primarily controls the coupling strength and, consequently, the 3-dB bandwidth and skirt characteristics. The core passband is captured by a 2-pole (Chebyshev-type) resonator network, while the DGS is modeled by an equivalent shunt ${L}_{\mathrm{DGS}}\text{, }{C}_{\mathrm{DGS}}$ branch that introduces the transmission zeros and reinforces stopband rejection, resulting in a quasi-elliptic response^31^.

The two resonators were modeled as parallel LC tanks using the following values:

$${\mathrm{L}}_{{1}} = {\text{ L}}_{{2}} = 0.{\text{439762 nH}},{\text{ C}}_{{1}} = {\text{ C}}_{{2}} = 0.{\text{1 pF}}$$

The capacitive coupling between the resonators was modeled by:

$${\mathrm{C}}_{{\mathrm{n}}} = 0.{\text{1 pF}}$$

Additionally, the dual DGS were modeled as a shunt LC branch composed of:

$${\mathrm{L}}_{{{\mathrm{DGS}}}} = 0.{\text{146587 nH}},{\text{ C}}_{{{\mathrm{DGS}}}} = 0.{\text{3 pF}}$$

The ADS-predicted ${S}_{11}$ and ${S}_{21}$ in Fig. 7c show good agreement with CST full-wave results, confirming that the model captures both the passband behavior and the DGS-induced transmission-zero mechanism.

## Result and discussion

The proposed 24 GHz bandpass filter demonstrates highly selective and stable performance, making it well-suited for short-range automotive radar and ADAS applications^32^. It features a sharp roll-off at both passband edges, effectively suppressing out-of-band interference and enhancing spectral efficiency. The filter offers a 450 MHz bandwidth centered at 24 GHz, aligning with the allocated ISM and automotive radar spectrum as it threats in Table 2.

**Table 2:** Specifications of the proposed ADAS BPF at 24 GHz.

Parameter	Value	Equation
Center frequency (fc)	23.970 GHz	${\mathrm{f}}_{\mathrm{c}}=({\mathrm{f}}_{\mathrm{low}}+{\mathrm{f}}_{\mathrm{high}})/2$
Bandwidth (3 dB)	0.4500 GHz	$\mathrm{BW}={\mathrm{f}}_{\mathrm{high}}-{\mathrm{f}}_{\mathrm{low}}$
Insertion loss (Min S12)	− 1.62 dB	$\mathrm{IL}\left({\mathrm{S}}_{12}\right)={\mathrm{minS}}_{12}\text{ within passband}$
Return loss (Min S11)	− 24.25 dB	$\mathrm{RL}\left({\mathrm{S}}_{11}\right)=\mathrm{min}\left({\mathrm{S}}_{11}\right)\text{over }\left[20:30\right]\mathrm{GHz}$
Roll-off rate	53.13 dB/GHz	$\mathrm{Roll}-\mathrm{off}=\frac{\left(-20\mathrm{dB}\right)-(-3\mathrm{dB})}{{\mathrm{f}}_{\mathrm{stop}}-{\mathrm{f}}_{\mathrm{passband}}}$
Selectivity ratio (quality factor)	53.23	$\mathrm{selectivity}({Q}_{L})= \frac{{\mathrm{f}}_{\mathrm{c}}}{\mathrm{BW}}$

**Fig. 8:**
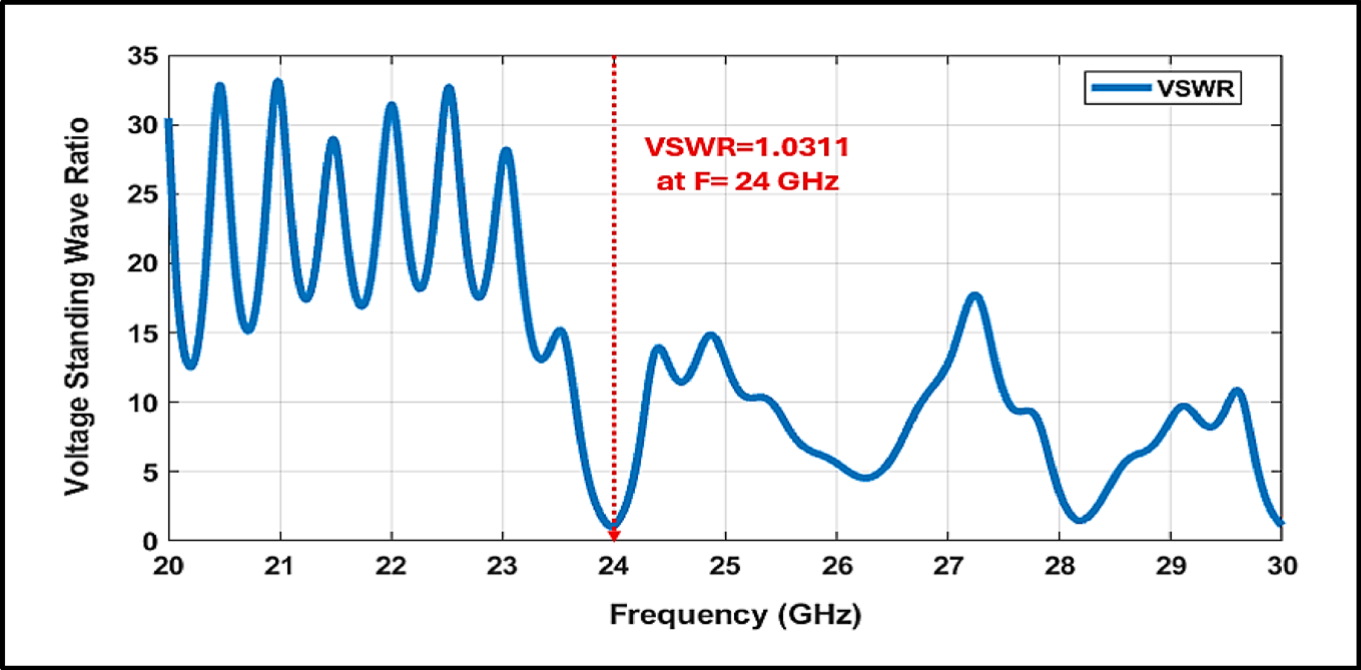
Simulated VSWR of the Proposed 24 GHz Bandpass Filter.

**Fig. 9:**
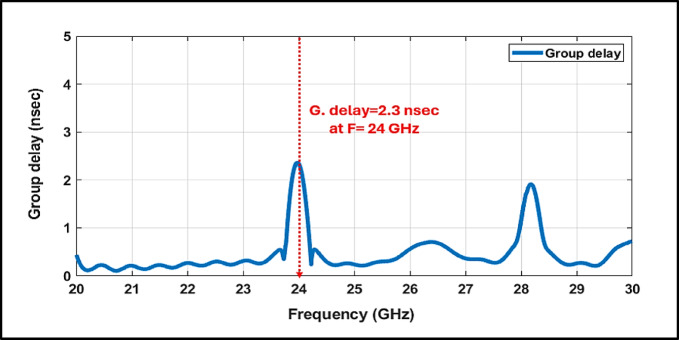
Simulated group delay response of the proposed 24 GHz bandpass filter.

Figure 8 illustrates the excellent impedance matching of the filter, demonstrated by a minimum VSWR of 1.03, which effectively reduces signal reflections and minimizes power loss. Furthermore, the group delay response is smooth and consistent, with a controlled peak of 2.3 ns at the center frequency and minimal variation across the band as in Fig. 9. This flat delay profile ensures high temporal resolution, which is essential for accurate target detection and velocity estimation.

## Thermal effects on the proposed filter geometry and response

To assess the thermal stability and reliability of the proposed 24 GHz bandpass filter under realistic environmental conditions, a detailed thermal analysis was conducted using CST Studio Suite. This investigation focused on evaluating the impact of temperature variation on the filter’s electromagnetic behavior, with particular emphasis on key performance parameters such as resonant frequency, ${\mathrm{S}}_{11}$, and ${\mathrm{S}}_{21}$. The simulation was carried out across a temperature range from 25 to 105 °C, reflecting the typical operating conditions encountered in automotive radar systems.

### Effect of thermal variation on S_11_ response

**Fig. 10:**
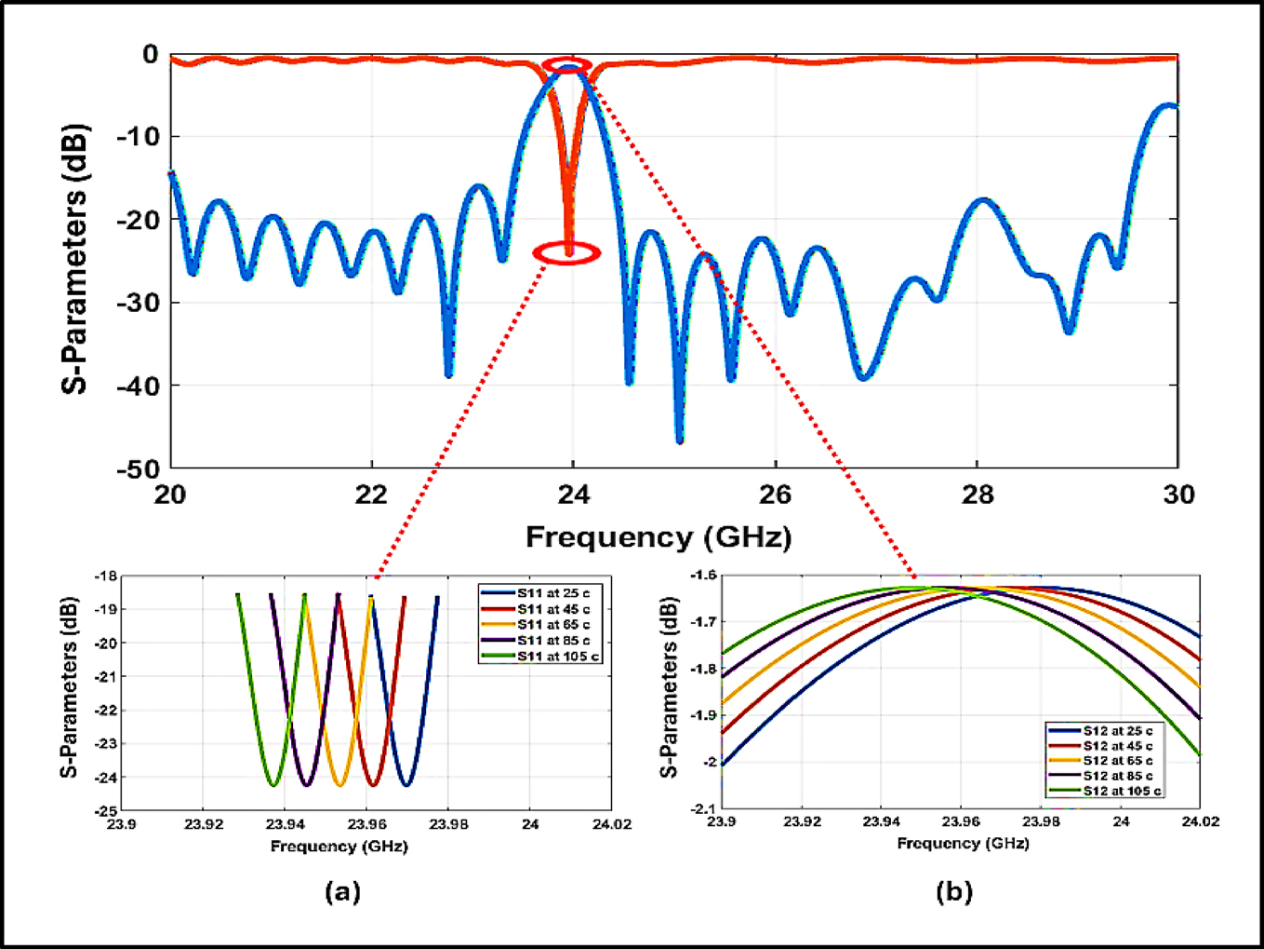
Thermal Effects on the proposed filter response of S_11_ and S_12_.

Figure 10a illustrates the effect of temperature variation on the ${\mathrm{S}}_{11}$ of the proposed 24 GHz bandpass filter across the thermal range of 25–105 °C^33^. As evident, the resonance identified by the minimum ${\mathrm{S}}_{11}$ progressively shifts toward lower frequencies with increasing temperature. This behavior is primarily attributed to the thermal expansion of the resonator’s physical structure, which results in an increase in the effective electrical length and consequently reduces the resonant frequency.

The thermal dependence of the resonator’s effective length L(T) is described by^34^:

7 $$L\left( T \right) = L_{0} \left( {1 + \beta \left( {T - T_{0} } \right)} \right),\,L\left( T \right) \propto \frac{1}{{F_{r} \left( T \right)}}$$

where L_0_ is the initial resonator length at the reference temperature T_0_, and $\beta$ is the linear thermal expansion coefficient (typically for copper $17 \times 10^{ - 6} \,^\circ {\mathrm{C}}^{ - 1}$). Accordingly, the temperature-dependent resonant frequency can be approximated by^35^:

8 $$F_{r} \left( T \right) = \frac{{f_{{\left( {r,0} \right)}} }}{{1 + \beta \left( {T - T_{0} } \right)}}$$

where ${f}_{(r,0)}$ is the resonant frequency at reference temperature T_0_ (usually 25 °C).

At 25°C, the filter demonstrates optimal impedance matching at approximately 23.97 GHz, achieving a return loss better than –24 dB. As the temperature rises to 105°C, the resonance frequency shifts to around 23.94 GHz with only slight degradation in return loss. Although the matching remains within acceptable limits, such thermally induced detuning could lead to spectral misalignment in temperature-sensitive radar systems, potentially affecting system performance.

### Effect of thermal variation on S12 response

Figure 10b presents the transmission coefficient S_21_ of the proposed 24 GHz bandpass filter under temperatures ranging from 25 to 105°C. The results clearly indicate a thermal-induced shift in the resonance frequency toward lower values as temperature increases, primarily due to the physical expansion of the resonator structure.

At 25°C, the filter achieves maximum transmission at approximately 23.97 GHz, with an insertion loss of –1.62 dB, indicating efficient power transfer within the passband. As the temperature increases to 105°C, the resonance shifts to 23.94 GHz, accompanied by a slight improvement in insertion loss to –2.01 dB, potentially attributed to reduced conductor losses at elevated temperatures.

Table 3 highlights the thermal influence on the proposed BPF performance, revealing a gradual shift in resonant frequency and slight variations in ${S}_{11}$ and ${S}_{21}$, while maintaining overall stability emphasizing the importance of incorporating thermal resilience into the design for reliable operation in critical radar environments. The ~ 30 MHz thermal shift (~ 0.12% of ${f}_{0}$) is far smaller than the 450 MHz bandwidth; thus, channel alignment remains intact and no special compensation is typically needed beyond routine radar front-end calibration.

**Table 3:** Thermal impact on filter characteristics.

Temperature (°C)	Resonant Freq (GHz)	Min S11 (dB)	Peak S12 (dB)	VSWR	Bandwidth (MHz)	Group Delay (ns)
25	23.97	− 24.25	− 1.62	1.031	450	2.3
45	23.961	− 23.82	− 1.71	1.045	446	2.35
65	23.954	− 23.47	− 1.82	1.062	442	2.41
85	23.946	− 23.01	− 1.92	1.078	437	2.48
105	23.94	− 22.63	− 2.01	1.095	432	2.54

## Fabrication and experimental verification

**Fig. 11:**
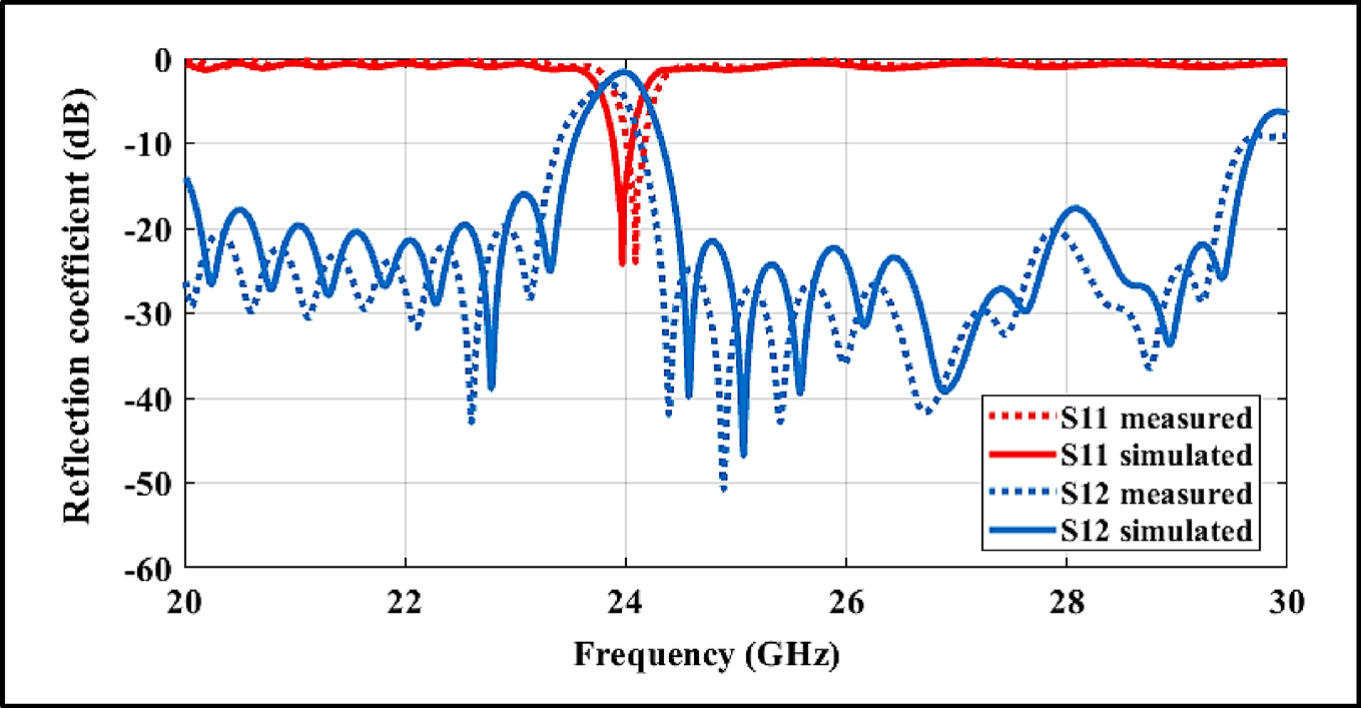
simulated and measured scattering parameters of BPF.

Figure 11 depicts the scattering parameters of the proposed 24-GHz band-pass filter over the 20–30 GHz frequency range. A sharp ${S}_{11}$ resonance is observed at approximately 24 GHz with a minimum of about − 22 dB, confirming robust in-band impedance matching. The passband centered around 24.2–24.6 GHz exhibits low insertion loss (≈ 0 to − 1 dB) with limited in-band ripple, indicating efficient power transmission suitable for automotive radar front-end requirements. Meanwhile, the stopband suppression exceeds ~ 35 dB at multiple off-resonant frequencies (around 23 GHz, 25–26 GHz, and near 29 GHz), demonstrating strong selectivity and effective rejection of adjacent-band interference.

The measured in-band insertion loss is primarily attributed to a combination of conductor loss associated with surface currents along the SIW cavity walls, via fences, and feeding lines at millimeter-wave frequencies, as well as dielectric loss of the substrate material at 24 GHz. Additional parasitic loss contributions arise from the CPW-to-SIW feeding transitions and the SMA-to-microstrip connector launches used during measurement. Minor discrepancies between the paired simulated and measured traces can therefore be reasonably explained by fabrication tolerances, surface roughness, and feed non-idealities, without altering the salient response characteristics required for 24-GHz automotive radar front ends. The measured insertion loss of 1.6–2.0 dB includes both intrinsic losses of the filter (conductor and dielectric losses, and CPW–SIW transition effects) and measurement-related contributions. All measurements were performed using end-launch 2.92-mm (K-type) coaxial connectors, whose launch and connector losses account for the slight increase relative to the simulated/intrinsic values.

**Fig. 12:**
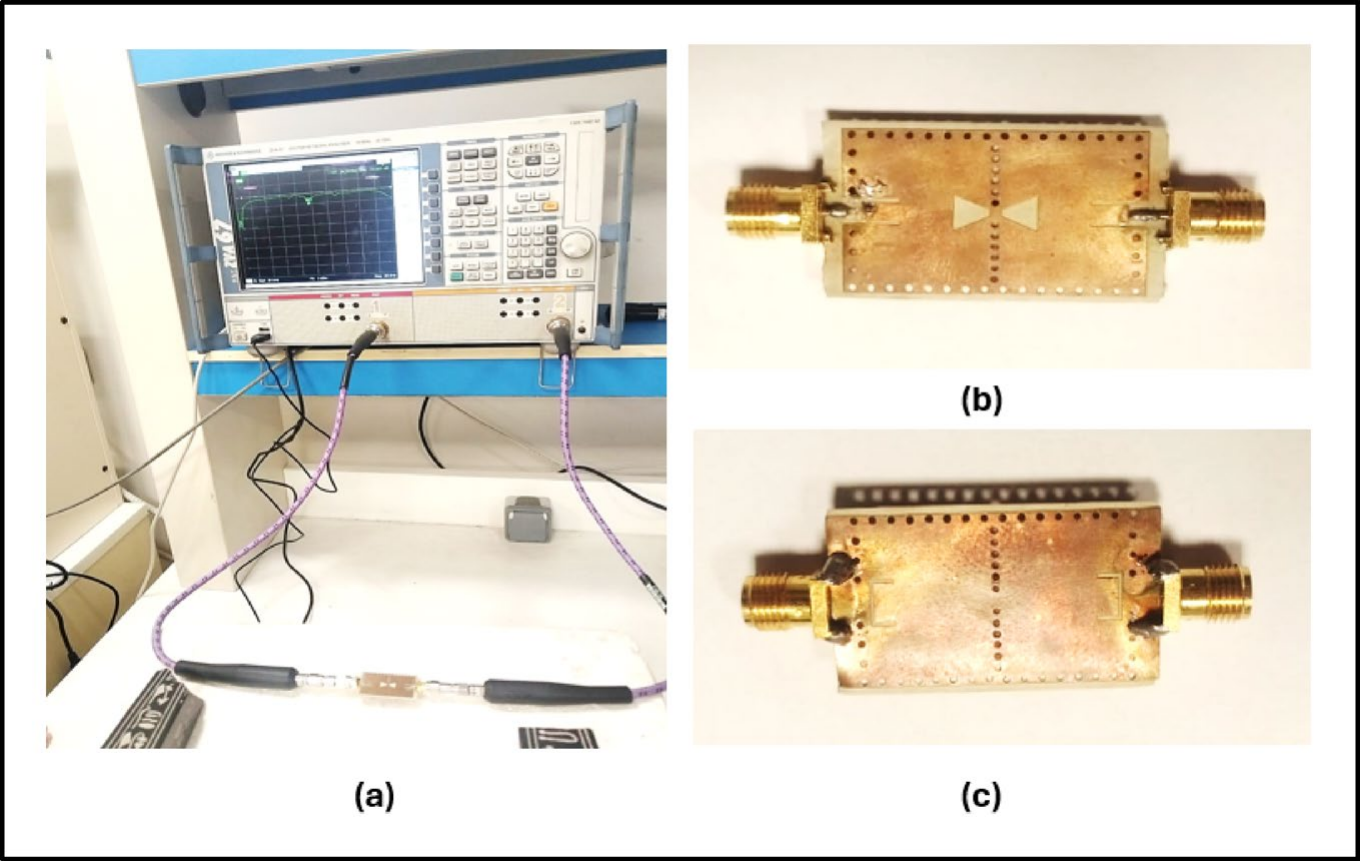
(**a**) Measurement Setup for the Fabricated 24-GHz SIW–DGS–CPW BPF Using an R&S ZVA-67 Vector Network Analyzer. (**b**) Top view of the BPF prototype. (**c**) Bottom view of the BPF prototype.

Figure 12 details the VNA-based measurement setup for the fabricated BPF prototype, where low-loss cables and well-matched connectors were used to ensure repeatable ${S}_{11}$/${S}_{12}$ readings and to substantiate the trends observed in Fig. 11.

Table 4 provides a concise comparison of recent BPF designs for automotive radar and ADAS systems. The proposed filter demonstrates superior selectivity and high performance, highlighting its advantage over existing counterparts in terms of performance and integration potential.

**Table 4:** Comparative analysis of recent BPF designs for automotive radar and ADAS applications.

References	Tech	Filter order	f_0_ (GHz)	Bandwidth (MHz)	Loaded Q ((Q_L_))	Size (${\lambda }_{g}\times {\lambda }_{g}$)	Insertion loss (dB)	Return loss (dB)
^4^	MEMS	First	24	450	53.3	(4.28 × 4.28)	1.82	> 16
^8^	SIW	Four	28.5	420	67.9	(1.90 × 2.28)	2.1	> 15
^9^	SIW	Four	23.92, 28.38	660, 880	36.2 / 32.3	(1.12 × 0.96)	1.4	> 14
^10^	SIW	Three	20.31	620	32.8	(1.60 × 0.93)	1.5	> 15
^14^	Rasorber	–	24	380	63.2	(0.80 × 1.44)	1.98	> 10
^16^	Hybrid	Four	24	530	45.3	(3.07 × 2.56)	1.72	> 15
This work	SIW	Second	24	450	53.3	(4.27 × 2.14)	1.62	> 24

## Conclusion

This research presented the design, equivalent circuit modeling, fabrication, and thermal analysis of a compact high-selectivity 24 GHz SIW-based bandpass filter for automotive radar and ADAS applications. The prototype achieves $RL>24{\hspace{0.17em}}\mathrm{dB}$, in-band IL of 1.6–2.0 dB, and a 450-MHz 3-dB bandwidth at $24{\hspace{0.17em}}\mathrm{GHz}$, with robust thermal stability (− 30 MHz over 25–105 °C). An equivalent circuit model was developed to validate the filter’s behavior, and the fabricated prototype confirmed the simulated performance. Thermal simulations from 25 to 105 °C showed a (~ 30) MHz frequency shift with minimal impact on ${S}_{11}$ and ${S}_{21}$, demonstrating the filter’s thermal resilience. The proposed filter offers a promising solution for radar front ends requiring spectral precision and environmental robustness. Future work will explore adaptive thermal compensation and in-situ calibration for enhanced reliability.

## Data Availability

All data supporting the findings of this study are available within the Article (including all reported simulated and measured results).
